# Encephalography cross-frequency coupling and brain alteration in amyotrophic lateral sclerosis

**DOI:** 10.1093/braincomms/fcaf192

**Published:** 2025-06-06

**Authors:** Cristina Benetton, Arnaud Preuilh, Mohammed Khamaysa, Maximilien Chaumon, Alexandra Lackmy-Vallée, Alper Er, Mélanie Pélégrini-Issac, Giorgia Querin, Caroline Rouaux, Pierre-François Pradat, Véronique Marchand-Pauvert

**Affiliations:** Sorbonne Université, Inserm, CNRS, Laboratoire d’Imagerie Biomédicale, LIB, Paris 75006, France; Sorbonne Université, Inserm, CNRS, Laboratoire d’Imagerie Biomédicale, LIB, Paris 75006, France; Sorbonne Université, Inserm, CNRS, Laboratoire d’Imagerie Biomédicale, LIB, Paris 75006, France; Sorbonne Université, Inserm, CNRS, Institut du Cerveau, ICM, Paris 75013, France; Sorbonne Université, Inserm, CNRS, Laboratoire d’Imagerie Biomédicale, LIB, Paris 75006, France; Sorbonne Université, Inserm, CNRS, Laboratoire d’Imagerie Biomédicale, LIB, Paris 75006, France; Sorbonne Université, Inserm, CNRS, Laboratoire d’Imagerie Biomédicale, LIB, Paris 75006, France; Sorbonne Université, Inserm, CNRS, Laboratoire d’Imagerie Biomédicale, LIB, Paris 75006, France; Assistance Publique—Hôpitaux de Paris (AP-HP), Département de Neurologie, Centre de référence pour la SLA, Hôpital Pitié-Salpêtrière, Paris 75013, France; Université de Strasbourg, Inserm UMRS 1329, Strasbourg Translational Neuroscience and Psychiatry (STEP), Centre de Recherche en Biomédecine de Strasbourg Strasbourg 67000, France; Sorbonne Université, Inserm, CNRS, Laboratoire d’Imagerie Biomédicale, LIB, Paris 75006, France; Assistance Publique—Hôpitaux de Paris (AP-HP), Département de Neurologie, Centre de référence pour la SLA, Hôpital Pitié-Salpêtrière, Paris 75013, France; Sorbonne Université, Inserm, CNRS, Laboratoire d’Imagerie Biomédicale, LIB, Paris 75006, France

**Keywords:** EEG, phase amplitude coupling, cortical excitability, MRI, cerebral atrophy

## Abstract

The diagnosis of amyotrophic lateral sclerosis requires identifying degeneration in both brain and bulbospinal motor neurons. However, detecting cortical dysfunction remains challenging, as peripheral symptoms often overshadow upper motor neuron signs. Although transcranial magnetic stimulation and MRI are valuable tools, transcranial magnetic stimulation is challenged as disease progresses but also at early stage in some patients, and brain MRI shows in most cohorts no significant change at the time of diagnosis. This emphasizes the need for neuromarkers facilitating detection of cortical dysfunction and longitudinal monitoring. EEG offers promising avenues. Accordingly, we recently identified altered theta-gamma phase–amplitude coupling in amyotrophic lateral sclerosis. The present study aimed to further explore phase–amplitude coupling in patients, focusing not only on theta and gamma bands but also on alpha and beta bands, and the link with handedness and brain structure. Resting-state EEG was recorded in 26 patients with amyotrophic lateral sclerosis and 26 age- and sex-matched controls, alongside anatomical and diffusion MRI. PAC was calculated between slow and gamma oscillations at five sensorimotor electrodes bilaterally. Grey and white matter integrity was evaluated through cortical thickness measurements and diffusion metrics along the corticospinal tract. Results revealed significantly decreased theta-gamma PAC in the dominant hemisphere of patients, without changes in band powers or other frequency couplings. MRI confirmed well-known handedness-related brain structural asymmetry in both groups, although it was less pronounced in patients. Specifically, diffusion metrics were altered in the most caudal segment (brainstem level) of the pyramidal tract within the dominant hemisphere in patients. These findings align with lateralized theta-gamma PAC alterations and the greater vulnerability of the dominant hemisphere to amyotrophic lateral sclerosis. No correlation was found between electrophysiological and diffusion metrics, likely because they are related to different mechanisms: PAC alteration being presumably linked to excitation/inhibition imbalance preceding upper motor neuron degeneration. Moreover, theta–gamma PAC was found to be particularly altered in patients with altered cognitive scores, consistent with previous findings in patients with mild cognitive impairment. Lastly, receiver operating characteristic analyses demonstrated that PAC outperformed diffusion MRI in diagnostic accuracy, underscoring its potential as a very sensitive marker of cortical dysfunction in amyotrophic lateral sclerosis. Although these results need validation in a larger cohort at different stages of the disease and across different forms (sporadic and familial), they confirm that PAC can detect cortical dysfunctions in amyotrophic lateral sclerosis.

## Teaser

Benetton *et al*. report that altered theta-gamma phase–amplitude coupling in amyotrophic lateral sclerosis is linked to handedness-related asymmetry, particularly in early-stage, slow-progressing and cognitively impaired patients. This alteration, associated with dominant hemisphere corticospinal changes, supports phase–amplitude coupling as a reliable marker of cortical dysfunction based on sensitivity and specificity analyses.

## Graphical Abstract

**Graphical Abstract:**
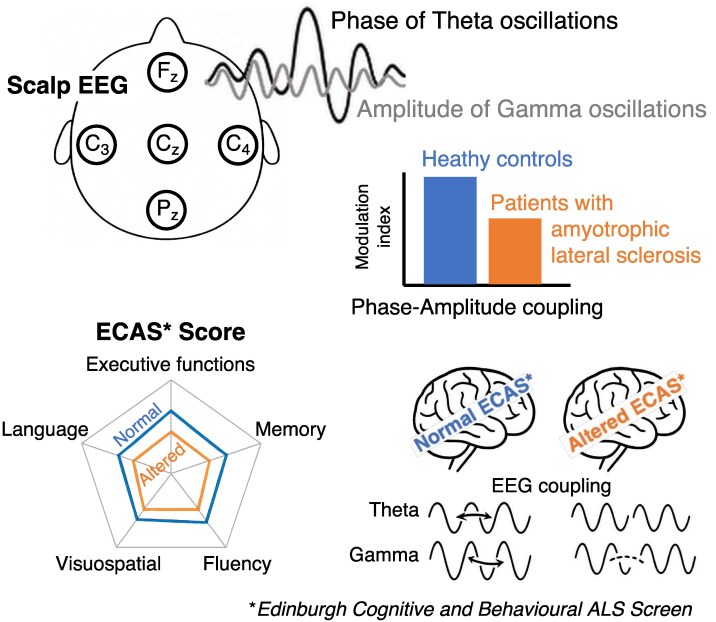

## Introduction

Amyotrophic lateral sclerosis (ALS) is a severe neurodegenerative disease that typically starts in adulthood, causing progressive muscle weakness, atrophy and eventually leading to death within 2–5 years, primarily due to respiratory failure.^1^ Its diagnosis hinges on identifying concurrent degeneration of upper motor neurons (UMN) in the cerebral cortex and lower motor neurons (LMN) in the brainstem and the spinal cord.^2^ However, confirming UMN signs is often challenging, as they are often masked by LMN signs.^3^ A diagnostic delay of one year is common, largely due to heterogeneous clinical presentations that are partly influenced by varying UMN and LMN involvement.^4,5^ With the advent of new treatment options,^6^ the importance of promptly identifying ALS has become increasingly crucial. As a result, there has been growing interest in developing biomarkers over recent decades for diagnosis and clinical trials.^7,8^

Cortical dysfunction, particularly cortical hyperexcitability, appears to be an early and inherent characteristic of both sporadic and familial forms of ALS. This phenomenon occurs before the onset of peripheral dysfunction and is associated with subsequent LMN impairment and degeneration.^9-11^ Inconsistent findings from brain neuroimaging studies are likely due to the heterogeneity of the disease. However, reduced cortical thickness and alteration of the corticospinal tract white matter are consistently reported.^12^ Alternatively, transcranial magnetic stimulation (TMS) provides robust and objective neuromarkers of UMN dysfunction in ALS.^13^ However, due to rapid disease progression, a significant number of patients become rapidly unable to undergo MRI or TMS, even within the first year after symptom onset. EEG is an interesting alternative because its implementation is independent of the patient's clinical status, and it has no contraindications. To date, EEG in ALS has been used for brain–machine interfaces,^14^ studying evoked potentials,^15^ and examining cortical connectivity.^16^ However, no quantitative metrics derived from basic EEG montages routinely used in clinics have been proposed to assess brain dysfunction.

A key feature of EEG oscillations is that their magnitude inversely correlates with frequency due to factors like cytoarchitecture, dendritic filtering, synaptic activity, time window and phase coherence, which affect signal power.^17^ Slow oscillations exhibit higher phase coherence compared with faster ones. Consequently, low-frequency signals have minimal impact on their own amplitude but can strongly modulate higher-frequency signals. This hierarchical interaction is often described by phase–amplitude cross-frequency coupling (PAC), which reflects communication across brain areas, specifically the influence of large-scale networks on the activity of smaller-scale, local circuits. PAC varies across frequency pairs and brain regions and relies on a proper balance between excitation and inhibition.^18-20^ Recently, we reported altered PAC in preclinical models of ALS and in sporadic patients.^21^ Our analysis was limited to the coupling between theta and gamma bands, which was found particularly depressed in the left sensorimotor cortex in patients. These first results support PAC as putative metric to assess cortical dysfunctions in ALS. However, this question needs further validation by comparing handedness-related dominant and non-dominant brain hemispheres and by combining PAC analysis with other, validated approaches. Moreover, PAC has been investigated across multiple frequency bands, particularly in Parkinson's disease, where beta–gamma coupling alterations have been reported,^22,23^ and in Alzheimer's disease, exhibiting broader PAC disruptions, including alpha–gamma coupling.^24,25^ To ensure a comprehensive assessment of our ALS patient cohort,^21^ we examined alpha–gamma and beta–gamma coupling to determine whether the observed coupling alterations were specific to a particular frequency pair (theta–gamma) or a widespread phenomenon.

The first objective of the present study was thus to evaluate PAC in frequency pairs beyond the theta–gamma bands. To do so, we first assessed the theta, alpha, beta and gamma bands, as variations in power can influence PAC. After confirming that there were no significant differences in power spectra between groups, we proceeded to compare PAC across different frequency pairs (theta–gamma, alpha–gamma and beta–gamma). The second objective was to investigate structural brain changes using validated ALS markers—specifically cortical thickness and white matter integrity—through multimodal MRI, to determine whether similar handedness-related asymmetries were present, as observed with PAC.^21^ Additionally, we examined PAC relationship with clinical phenotype by assessing its association with cognitive impairment. Lastly, we evaluated its performance in distinguishing patients from controls.

## Materials and methods

### Ethics

This study was conducted in compliance with the latest revision of the Declaration of Helsinki.^26^ It serves as an ancillary part of a research project on cortical integration in ALS, with experimental procedures approved by the Inserm ethics committee (clinical research sponsor; protocol C17–70). The study received authorization from the French Ethics Committee Ouest II Angers (#18.07.11.57804 2018/58; ID RCB 2018-A00789–52) and was registered on ClinicalTrials.gov (NCT 03694132).^27^ All participants provided written informed consent before inclusion.

### Participants

Fifty-two participants were included: 26 patients with sporadic ALS (nine females, mean age ± 1 standard error, 61.3 ± 2.4 years old) and 26 age- and sex-matched healthy controls (eight females, 63.4 ± 2.1 years old). The main inclusion criteria for patients were probable or definite ALS diagnosis according to the El Escorial criteria,^28^ confirmed by neurologists of the Paris ALS Referent Centre (PF.P and G.Q; Pitié-Salpêtrière Hospital, Paris, France), and absence of medical conditions associated with peripheral neuropathy. According to the full study,^27^ they had to exhibit no or only limited motor dysfunction in hands, with muscle strength evaluated by manual muscle testing and rated using the cumulative Medical Research Council scores. Table 1 summarizes the key clinical features, including the Medical Research Council scores on right and left hand, the total score to ALS Functional Rating Scale—revised (ALSFRS-r),^29^ the ALS-specific and total score to Edinburgh Cognitive and Behavioural ALS Screen (ECAS)^30,31^; all patients but one were treated by riluzole. Healthy controls were enrolled if they had no history of neurologic condition. Handedness was assessed using the Edinburgh Handedness Inventory^32^; 23 patients (Table 1) and 24 controls were right-handed.

**Table 1:** Clinical features

Patient	Handedness	Duration	Onset	MRC_right_	MRC_left_	ALSFRS-r	ECAS_specific_	ECAS_total_	Form
1	Right	60	LL (left)	8	6	37	41	64	PLMN
2	Left	36	B	9	9	34	73	99	PLMN
3	Right	18	LL	9	9	41	90	124	PUMN
4	Right	45	LL (right)	8	6	36	79	108	Classical
5	Right	10	UL (left)	10	4	44	62	91	PUMN
6	Left	15	LL (left)	10	10	44	82	114	PUMN
7	Right	14	UL (right)	8	8	43	78	107	Classical
8	Left	36	UL (right)	10	10	40	80	114	PLMN
9	Right	15	LL (left)	9	9	42	-	-	PLMN
10	Right	8	UL (right)	7	8	36	67	102	PLMN
11	Right	8	B	9	9	34	44	66	Classical
12	Right	8	LL (right)	8	9	43	83	118	PLMN
13	Right	76	LL (left)	10	10	41	63	95	PUMN
14	Right	37	LL (right)	8	10	34	83	112	PLMN
15	Right	22	UL (left)	10	10	38	89	123	PUMN
16	Right	11	B	10	10	43	69	94	PUMN
17	Right	6	B	10	10	47	81	114	Classical
18	Right	3	LL (left)	10	10	40	92	122	PLMN
19	Right	43	B	9	10	39	64	91	PUMN
20	Right	31	UL (right)	6	8	35	79	109	Classical
21	Right	20	UL (left)	10	9	47	77	111	PLMN
22	Right	9	LL (left)	10	10	40	88	123	PLMN
23	Right	7	UL (left)	10	10	43	80	109	PLMN
24	Right	9	UL (right)	5	8	35	77	109	PLMN
25	Right	8	LL (right)	8	10	40	89	120	PUMN
26	Right	7	LL (left)	8	9	38	87	118	PUMN

Patients by order of inclusion. Handedness: motor lateralization. Duration: time since first symptoms (months). Onset: first symptoms in lower limb (LL), upper limb (UL) or bulbar muscles (B), with the side of first symptoms between brackets when lateralized. MRC score on the right and left hand: summation of score for abductor pollicis brevis (APB) and flexion of finger III at the distal phalanx (maximum score on each side = 10 indicating normal strength). ALSFRS-r, ALS Functional Rating Scale—revised (maximal score = 48 when all items are unaltered). Edinburgh Cognitive and Behavioural ALS Screen (ECAS) with ALS-specific score (ECASspecific; score > 77/100 = no cognitive impairment) and total score (ECAS_total_; score > 105/136 = no cognitive impairment). Form: predominant upper motor neuron involvement (PUMN), predominant lower motor neuron involvement (PLMN), or classical form (with equal involvement of UMN and LMN). All patients but one were on riluzole (#5). Part of the clinical features are in Scekic-Zahirovic *et al*.^21^

### Experimental procedures and signal processing

All experiments were conducted at the Center for NeuroImaging Research (CENIR) of the Brain Institute (ICM; Pitié-Salpêtrière Hospital). EEG and MRI scans were conducted on the same day, with EEG performed in the morning and MRI in the afternoon. One patient and one control were unable to complete the MRI scan due to technical issues and discomfort, respectively.

### EEG

EEG cap with 74 Ag/AgCl annular electrodes (EasyCap GmbH, Herrsching, Germany; Nuwer 2018)^33^ was positioned according to the international 10–20 system, ensuring that the Cz electrode was aligned with the anatomical vertex point. Water-soluble conducting gel was applied to each electrode, and impedance was individually checked (∼5–10 kΩ) before acquisition. Single-use pre-gelled Ag/AgCl electrodes (Ambu® Neuroline 720, Ballerup, Denmark) were placed on the right earlobe for the reference electrode, on the left scapula for the ground electrode, above and below the right eye for electrooculogram (EOG), and on the right clavicle and the lower left part of the abdomen for electrocardiogram (ECG). During recordings, participants were instructed to remain relaxed and minimize movement (resting-state EEG). Our previous analysis revealed depressed theta–gamma PAC in ALS, especially when EEG was collected with eyes closed.^21^ Thus, this study focuses solely on the results obtained during the 5-minute period when all data were recorded with the participants’ eyes closed.^25^

**Figure 1:**
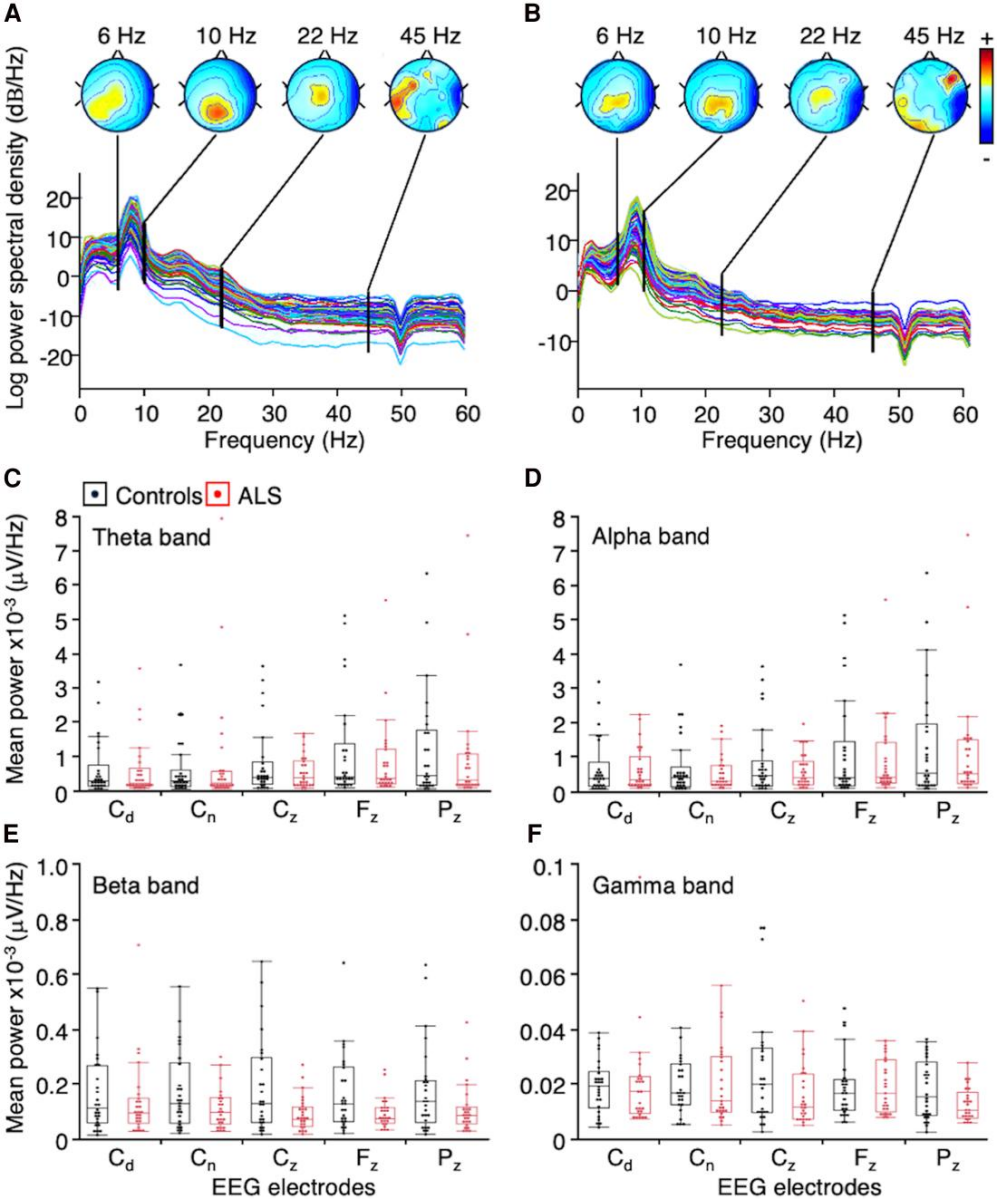
**Power spectral analysis.** (**A and B**) Topography of the signal power at 6 (within the theta band), 10 (within the alpha band), 22 (within the beta band) and 45 Hz (within the gamma band) in one healthy control (**A**) and one patient with amyotrophic lateral sclerosis (ALS) (**B**). The XY plot below represent the log_10_ power (dB/Hz) plotted against the frequency (Hz) for all the 74 channels of the EEG cap. The colour scale is adjusted for each topographical map individually so as to span the entire range of the signal shown with black bars on the spectrum. (**C-F**) Box plots representing the distribution of the mean power in theta (**C**), alpha (**D**), beta (**E**) and gamma bands (**F**) at the level of electrodes over the primary sensorimotor areas corresponding to C_3_ and C_4_ electrodes, recoded according to the handedness-related dominant (C_d_) and non-dominant hemisphere (C_n_), C_z_, F_z_ and P_z_ (according to the EEG 10–20 international montage). The central box represents the interquartile range (IQR). The bottom and top edges of the box correspond to the first quartile (Q1) and third quartile (Q3), respectively. The line inside the box represents the median (Q2). The lines extending from the top and bottom of the box (whiskers) represent the range of the data outside the IQR (1.5×IQR from Q1 and Q3). Each point represents data from an individual (controls in black and patients with ALS in red), with points outliers appearing outside the whiskers’ limits. *N* = 26 controls and 26 patients with ALS. Linear mixed model: Group (Controls versus ALS), *F*(1,48.95) = 0.0199, *P* = 0.88.

EEG was processed using EEGLAB 2021.1, an open-source toolbox running in MATLAB® (R2019b; The Mathworks, Inc., Natick, MA, USA).^34^ The data were first down-sampled (400 Hz), re-referenced (average reference)^35^ and filtered (50-Hz notch filter, 2–60 Hz bandpass). Channels with excessive noise and artefacts were initially rejected to ensure data accuracy. Then, independent component analysis was applied and the repetitive non-neural components (electromyogram -EMG- from scalp and facial muscles, EOG and ECG) were removed from EEG.^36-38^ The denoised time-series were visually inspected again to remove any segments containing irregular artefacts (e.g. irregular head/body movements, discontinuities caused by filtering operations) that were not captured by independent component analysis. Next, EEG signal decomposition was performed using the fast Fourier transform to analyse the power within distinct frequency bands, namely theta (4–8 Hz), alpha (8–13 Hz), beta (13–30 Hz) and gamma (30–60 Hz). Power spectral density was then calculated for each band, and the spatial distribution of power across the scalp was visualized using topographical mapping techniques (Fig. 1A and B). Our analyses focused on the electrodes C_3_, C_4_, C_z_, F_z_, and P_z_, which predominantly capture signals of interest originating from the centro-parietal regions. These electrodes were also selected based on their relevance to the targeted neural activity and their coverage of the primary sensorimotor areas of interest in ALS.

**Figure 2:**
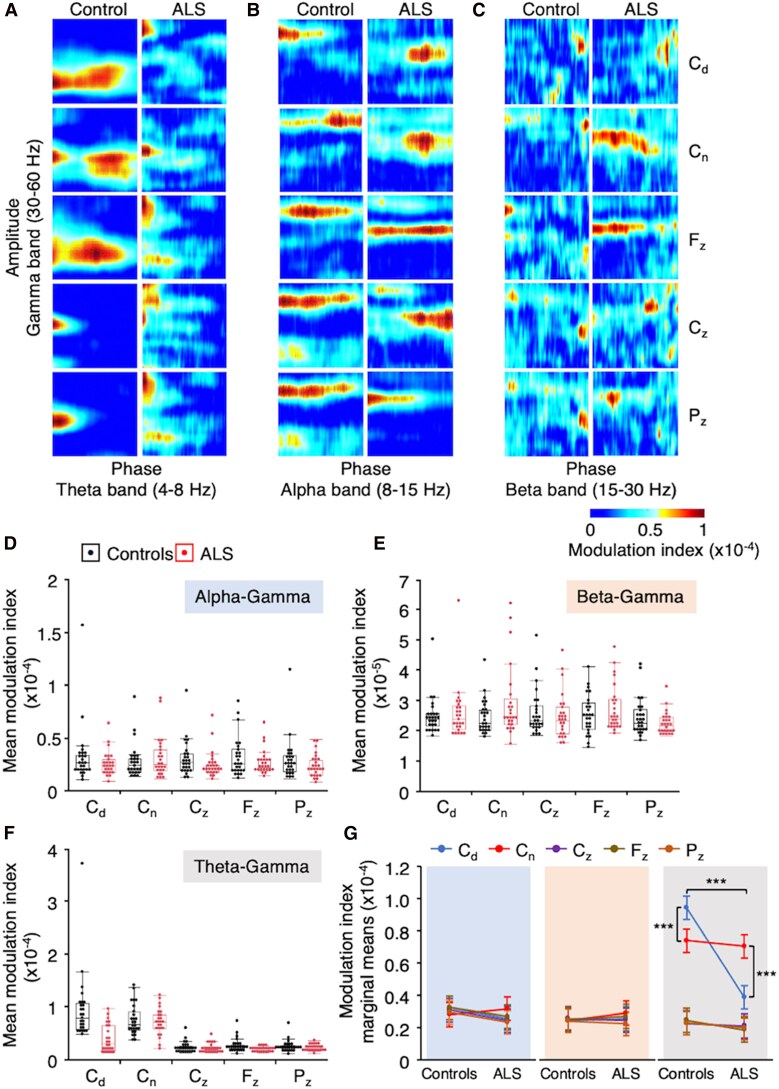
**PAC between frequency pairs in handedness-related dominant and non-dominant sensorimotor cortex.** (**A–C**) Phase–amplitude comodulogram computed from EEG data in one control (left columns) and one patient with amyotrophic lateral sclerosis (ALS; right columns), illustrating cross-frequency coupling interactions between low-frequency phase (theta in **A**, alpha in **B** and beta in **C**) and high-frequency amplitude (gamma) oscillations. The heatmap shows the coupling strength, where higher values (in red) indicate strong modulation of high-frequency amplitude by low-frequency phase. (**D-F**) Box plots as in Fig. 1, representing the distribution of the mean modulation index of phase–amplitude (PAC) between alpha-gamma (**D**), beta-gamma (**E**) and theta-gamma bands (**F**), in controls (black) and ALS (red) at the level of the five electrodes of interest; C_3_ and C_4_ electrodes being recoded according to the handedness-related dominant (C_d_) and non-dominant (C_n_) hemispheres. (**G**) Marginal means estimated from a repeated measure linear mixed model which, provide the average predicted values of the alpha-gamma (blue zone), the beta-gamma (orange zone) and the theta-gamma (grey zone) PAC modulation index in controls and patients with ALS, at the level of C_d_ (blue dots and lines), C_n_ (red dots and lines), C_z_ (purple dots and lines), F_z_ (brown dots and lines) and P_z_ (dark orange dots and lines). Error bars indicate the standard errors of the estimates. *N* = 26 controls and 26 patients with ALS. Repeated measure linear mixed model: Group (Controls versus ALS), *F*(1, 51.07) = 4.4071, *P* < 0.05. *** *P* < 0.0001 after *post hoc* comparisons.

PAC analysis was conducted using the modulation index method, originally introduced by Tort and colleagues in 2008.^39^ This method is widely regarded as a preferred measure of PAC due to its robustness and reliability under diverse conditions. Unlike techniques that depend on specific amplitude features (e.g. mean or peak amplitude), which are sensitive to outliers, the modulation index quantifies PAC by assessing the entire distribution of amplitudes across phase bins. By incorporating Kullback–Leibler divergence to compare the empirical PAC distribution against a uniform reference, this approach enhances resilience to noise and non-stationarities in real-world data. Comparative studies have consistently demonstrated the superior performance of the modulation index in both simulated and empirical datasets, compared with other methods.^40,41^ The modulation index was computed for each phase–amplitude pair (theta–gamma, alpha–gamma, beta–gamma) in each individual, focusing on the five EEG electrodes of interest (C_3_, C_4_, C_z_, F_z_, P_z_). Each frequency band was divided into 100 evenly spaced bins, ensuring fine resolution for the PAC analysis across the entire frequency ranges. The modulation index for each bin was visualized using the jet colormap, generating comodulograms (Fig. 2A and C). Finally, the mean modulation index was extracted from each comodulogram for group-level analyses.

### MRI

MRI sessions were conducted using a 3-T MRI scanner Magnetom Verio syngo MR B17 (Siemens Healthcare, Erlangen, Germany) equipped with a 32-channel head coil: T1-weighted sequence (MP-RAGE sequence; TR: 2300 ms, TI: 900 ms, TE: 3.23 ms, voxel size: 1×1 × 1 mm^3^, in-plane matrix size: 256×256 voxels, flip angle: 9°, 192 slices), and diffusion-weighted sequence optimized for high angular resolution diffusion imaging (two sets with reverse phase encoding, of 60 diffusion directions each, b-value: 1500 s/mm^2^, voxel size: 2.3×2.3×2.3 mm^3^).

The forward deformation field between the native T1-weighted anatomical image and the MNI152 T1-weighted template was estimated using CAT12.^42,43^ This field was used to normalize the anatomical image to the MNI standard space. Within this space, T1 images were segmented using the DARTEL algorithm to extract grey matter, white matter and cerebrospinal fluid.^44^ Subsequently, the cortical thickness of the primary sensorimotor cortex was measured (aal3 atlas).^45-47^

**Figure 3:**
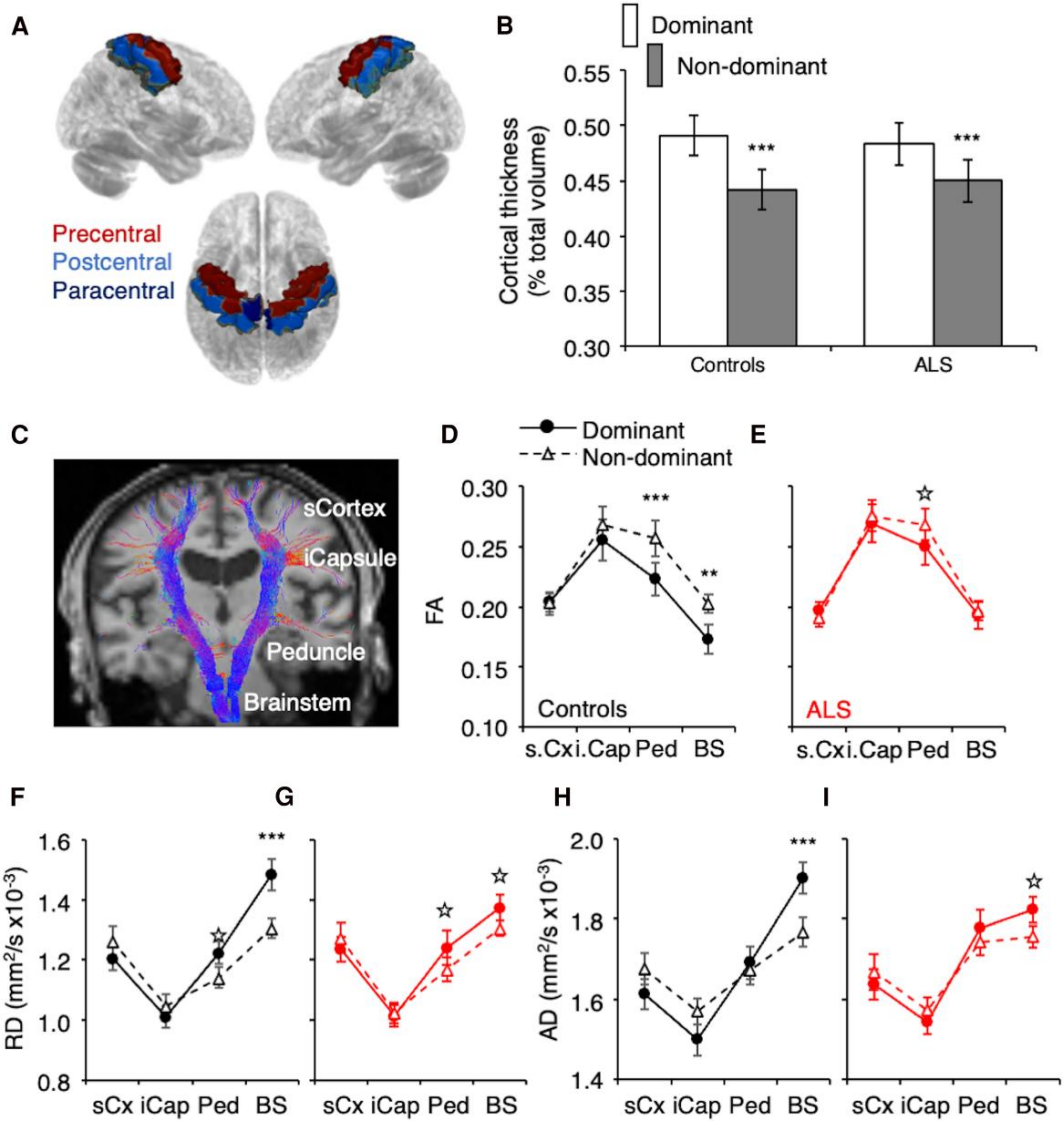
**Structural brain changes**. (**A**) Cortical thickness was measured in the precentral (red), postcentral (blue), and paracentral regions (dark blue), which correspond to areas covered by the C_n_ and C_d_ electrodes (images extracted from one control). (**B**) The grey matter volume in each region was normalized to the total intracranial volume of each individual. The bars represent the mean normalized volumes in the three regions of interest (±1 standard error) for the dominant (white bars) and non-dominant hemispheres (grey bars) in controls (left) and patients with amyotrophic lateral sclerosis (ALS; right). Repeated measure linear mixed model: Brain hemispheres (dominant versus non-dominant), *F*(1,50) = 66.8358, *P* < 0.0001. (**C**) Tractography of the right and left corticospinal tract in one patient with ALS overlaid on a T1-weighted MRI coronal view. Diffusion metrics were calculated near the cerebral cortex (sub-cortex, s.Cortex), at the level of the internal capsule (i.Capsule), the cerebral peduncles (Peduncle) and the brainstem (Brainstem). (**D** and **E**) Mean fractional anisotropy (FA; ±1 standard error) for the dominant (filled circles, solid lines) and non-dominant corticospinal tracts (triangles; dashed lines) for the control group (black, **D**) and the ALS group (red, **E**) across the four levels of interest (s.Cx: sub-cortex level, i.Cap: internal capsule, Ped: cerebral peduncles, and BS: brainstem). (**F** and **G**) Mean radial diffusivity (RD, mm^2^/s × 10^−3^) for the control group (**F**) and ALS group (**G**), same legend as in DE. (**H** and **I**) Mean axial diffusivity (AD, mm^2^/s × 10^−3^) for the control group (**H**) and ALS group (**I**), same legend as in **D** and **E**. Repeated measure linear mixed model: Brain hemispheres (dominant versus non-dominant): (i) FA: *F*(1,42.12) = 12.6165, *P* < 0.001, (ii) RD: *F*(1,47.26) = 8.8697, *P* < 0.01 and (iii) Ad: *F*(1,47.24) = 1.053, *P* = 0.31. *N* = 26 controls and 26 patients with ALS. *P < 0.05, **P < 0.001, ***P < 0.0001 after *post hoc* comparisons.

Diffusion MRI preprocessing included distortion correction with FSL top-up tool, eddy current correction and bias field correction using the N4 algorithm from ANTs library.^48-50^ A brain mask was generated using FSL's Brain Extraction Tool (BET). Diffusion tensor fitting was performed with FSL's DTIfit, resulting in diffusion tensor imaging (DTI) maps of fractional anisotropy (FA), and axial and radial diffusivities (Ad and RD, respectively). The T1-weighted images were registered to the diffusion space using FreeSurfer.^51-53^ Fibre orientation distributions were then estimated from diffusion data using spherical deconvolution, and the corticospinal tract was delineated using probabilistic streamline tracking using MRtrix3,^54^ with seed regions defined by the JHU DTI-based white matter atlas.^55^ Weighted diffusion metrics were indeed calculated along the corticospinal tract, across different brain regions (subcortical, internal capsule, cerebral peduncle, brainstem; Fig. 3C).

### Statistical analysis

Statistical analyses were undertaken with JMP® Pro 17 (SAS Inc., Cary, NC, USA). The results were considered statistically significant only if *P* < 0.05. Mean values are indicated ± 1 standard error. Homoscedasticity (Levene test) and normality (Shapiro–Wilk test) were tested, and significant outliers were detected using the inter-quantile range (IQR) method. Effect size was measured using (i) Cohen's d for two-condition comparisons,^56,57^ (ii) f^2^ for linear mixed models (LMM)^58^ and (iii) Cramér's V for Chi2 and receiver operating characteristic (ROC) analysis.^59^ Univariate analyses were conducted using LMM on repeated measures (RM-LMM) to compare groups and conditions with respect to the following metrics (values for each brain hemisphere or the difference between dominant and non-dominant hemispheres): (i) signal power (because variations in power can influence PAC),^60^ (ii) PAC modulation index (to compare between different frequency pairs), (iii) cortical thickness and (iv) diffusion metrics (to evaluate brain structures and possible asymmetrical changes). Random effects included random intercepts for participants and random slopes for conditions to account for individual variability in responses across conditions. The models incorporated fixed effects for group (a between-subject factor: controls versus ALS) and conditions (within-subject factors: electrode sites, power bands, frequency pairs, brain hemispheres, and rostro-caudal sites). *Post hoc* pairwise comparisons of marginal means were performed using Student *t*-tests. In patient group, possible links between functional (EEG) and ECAS score or structural (MRI) measures were tested using Pearson's correlation analyses. Lastly, ROC curve analysis was performed to compare the performance of EEG and MRI metrics to stratify patients from controls. For clarity, the statistical tests and the parameters included in each model are explicitly detailed in the Results section.

## Results

### Specific theta–gamma PAC alteration in the dominant sensorimotor cortex

#### Comparison of power spectral density

We first compared the power spectral density in each band, given its influence on PAC.^60^ Fig. 1A and B show the results from the seventy-four EEG electrodes for one control (A) and one ALS patient (B), along with the topography of the power within the four bands of interest. In both subjects, we observed a peak within the theta–alpha band and a smaller peak in the beta band; the gamma band peak is barely visible due to the scale. The box plots in Figs 1C–F display the mean power in each band at the five EEG electrodes of interest, across each group. For the C_3_ and C_4_ channels, data were aligned according to the handedness-related brain asymmetry; C_3_ becoming C_d_ (d for dominant hemisphere) and C_4_, C_n_ (for non-dominant hemisphere) in right-handers, and *vice versa* in left-handers. Significant outliers were identified using IQR method: one in the alpha band (C_n_; patient#16, Table 1), three in the beta band (C_n_, F_z_, P_z_; patient#16), five in the gamma band (C_d_, C_n_, F_z_, C_z_; patient#16; F_z_, control#16). LMMs were calculated to examine differences in power across groups (controls versus ALS). Power was analysed with respect to handedness-related channels (C_d_, C_n_, C_z_, F_z_, P_z_), and frequency bands (theta, alpha, beta and gamma). The model (adjusted r^2^ = 0.94) revealed no significant differences between groups [*F*(1,48.95) = 0.0199, *P* = 0.88, d = 0.10], and no significant interaction between groups and handedness-related channels [*F*(4164.1) = 0.8536, *P* = 0.49, f^2^ = 0.30] nor between groups and frequency bands [*F*(3149.9) = 0.7287, *P* = 0.53, f^2^ = 0.30]. Taken together, the data show that the present cohort of ALS patients does not present with altered power spectral density compared with healthy subjects.

#### PAC across frequency pairs

PAC was estimated across frequency pairs in each participant. Figure 2A–C shows how the phase of low-frequency oscillations (theta in Fig. 2A, alpha in Fig. 2B and beta in Fig. 2C) modulated the amplitude of gamma oscillations in single participants from the group of controls (left columns) and of ALS (right columns). The mean modulation index extracted from comodulograms in each participant is displayed in Fig. 2D–F, showing its distribution across groups at the five EEG channels of interest for each frequency pair. RM-LMM (no significant outliers; adjusted r^2^ = 0.74) revealed a significant difference between groups [controls versus ALS; *F*(1,51.07) = 4.4071, *P* < 0.05, d = 0.24] and a significant interaction among groups, handedness-related channels (C_d_, C_n_, C_z_, F_z_, P_z_), and frequency pairs [theta–gamma, alpha–gamma, beta–gamma; *F*(8,661.4) = 10.9097, *P* < 0.0001, f^2^ = 2.01]. *Post hoc* comparisons (Fig. 2G) showed significant differences in theta-gamma PAC between groups at the C_d_ electrode (*P* < 0.0001, d = 1.13) and between C_d_ and C_n_ within both groups (*P* < 0.0001, d = 0.41 in controls and 1.23 in ALS). The remaining pairwise comparisons (controls versus ALS for each frequency pair at the five electrodes, and C_d_ versus C_n_ for each frequency pair) were not significant (0.16 < *P* < 0.92). Collectively, the data indicate that (i) alpha–gamma and beta–gamma PAC were comparable between the two groups, (ii) in healthy controls, theta–gamma PAC was stronger in the dominant sensorimotor area as compared to the non-dominant area and (iii) in the ALS group, this coupling was significantly reduced in the dominant sensorimotor area.

Given the less lateralized brain organization in left-handers, who are typically excluded from neuroimaging studies,^61^ the model was recalculated without them (two controls and three patients; Table 1). However, the interaction between groups, channels and frequency pairs yielded similar results.

### Changes in diffusion metrics in the dominant brain hemisphere

#### Handedness-related symmetry in brain structures

Cortical thickness was assessed at the level of precentral, postcentral and paracentral areas (Fig. 3A) i.e. in the regions corresponding to the areas covered by the EEG electrodes of interest. RM-LMM (no significant outliers; adjusted r^2^ = 0.84) revealed significant differences between dominant and non-dominant hemispheres [*F*(1,50) = 66.8358, *P* < 0.0001, d = 0.92], and no significant interaction between groups and hemispheres [Controls versus ALS; *F*(1,50) = 3.2708, *P* = 0.07, f^2^ = 0.07]. *Post hoc* comparisons revealed significant differences between both hemispheres in each group (Controls: *P* < 0.0001, d = 1.14; ALS: *P* < 0.0001, d = 0.69; Fig. 3B).

Figure 3C illustrates a T1-weighted MRI in one ALS patient with superimposed corticospinal tract tractography. Diffusion metrics were calculated at four different levels along the corticospinal tract: just below the cortex (s.Cortex), at the level of the internal capsules (i.Capsule), of the cerebral peduncles (Peduncle) and of the brainstem in each participant, on the dominant and non-dominant hemispheres. RM-LMM including group (Controls versus ALS), hemisphere (Dominant versus Non-dominant) and level (s.Cortex versus i.Capsule versus Peduncle versus Brainstem) as fixed effects were calculated for each diffusion metrics (FA, AD, RD; no significant outliers were detected). Results for the different diffusion metrics:

- FA (adjusted r^2^ = 0.92): significant differences between dominant and non-dominant hemispheres [*F*(1,42.12) = 12.6165, *P* < 0.001, d = 0.18], between levels [*F*(3140.4) = 53.9984, *P* < 0.001, f^2^ = 0.16], and significant interaction between both factors [*F*(3139.1) = 5.7476, *P* < 0.001, f^2^ = 0.16]. *Post hoc* comparisons: significant differences between both hemispheres in controls at the most caudal levels of the corticospinal tracts (peduncle: *P* < 0.0001, d = 0.47; brainstem: *P* < 0.001, d = 0.59) and only at the peduncle level in ALS (peduncle: *P* < 0.05, d = 0.27; brainstem: *P* = 0.41, d = 0.07).
- RD: (adjusted r^2^ = 0.82): significant differences between hemispheres [*F*(1,47.26) = 8.8697, *P* < 0.01, d = 0.14], between levels [*F*(3140) = 103.0256, *P* < 0.001, f^2^ = 0.46] and significant interaction between both factors [*F*(3139.8) = 12.6745, *P* < 0.001, f^2^ = 0.47]. *Post hoc* comparisons: significant difference between both hemispheres at the most caudal levels of the corticospinal tracts in both groups (Controls: peduncle, *P* < 0.05, d = 0.45; brainstem, *P* < 0.0001, d = 0.83; ALS: peduncle, *P* < 0.05, d = 0.29; brainstem, *P* < 0.05, d = 0.41).
- AD: (adjusted r^2^ = 0.71): no significant global effect of handedness-related hemisphere [*F*(1,47.24) = 1.053, *P* = 0.31, d = 0.04], but significant differences between levels [*F*(3140.1) = 69.8724, *P* < 0.001, f^2^ = 0.31], and significant interaction between both factors [*F*(3141.2) = 9.7569, *P* < 0.001, f^2^ = 0.33]. *Post hoc* comparisons: significant difference between hemispheres at the brainstem level in both groups (Controls: *P* < 0.0001, d = 0.71; ALS *P* < 0.05, d = 0.47).

Taken together, these results indicate a handedness-related brain asymmetry in diffusion metrics at the most caudal regions of the corticospinal tract, including the peduncles and brainstem levels, in both groups.

### Differences in diffusion metrics between brain hemispheres at brainstem level

In all the tested models, neither the overall effect of the group factor (Controls versus ALS, 0.60 < *P* < 0.89) nor its interaction with other factors (group × hemisphere × level, 0.20 < *P* < 0.64) showed statistical significance. However, the asymmetry between both hemispheres at the most caudal levels of the corticospinal tract (cerebral peduncles and brainstem) was less pronounced in ALS compared to controls (Fig. 3D–I). *Post hoc* analyses revealed significant differences between groups on the dominant side for RD at the brainstem level (Student's *t*-test, *P* < 0.05, d = 47) and for AD at the peduncle level (Student's *t*-test, *P* < 0.05, d = 41). To further investigate the asymmetry in DTI metrics between hemispheres, the differences in FA, AD and RD between the dominant and non-dominant hemispheres were calculated for each participant at the levels of cerebral peduncles and brainstem. The models revealed a significant group effect only for the differences in FA [r^2^ = 0.38, *F*(1,47) = 6.2907, *P* < 0.05, d = 0.46] but not for AD [r^2^ = 0.54, *F*(1,47) = 0.0288, *P* = 0.866, d = 0.04] not for RD [r^2^ = 0.56, *F*(1,47) = 0.3714, *P* = 0.54, d = 0.14]. Overall, the diffusion MRI data revealed handedness-related brain structural asymmetry in both groups. However, in the ALS group, this asymmetry was less pronounced or absent, particularly for FA in the distal portion of the corticospinal tract (brainstem level).

## Clinical implications

### Link with clinical phenotype

**Figure 4:**
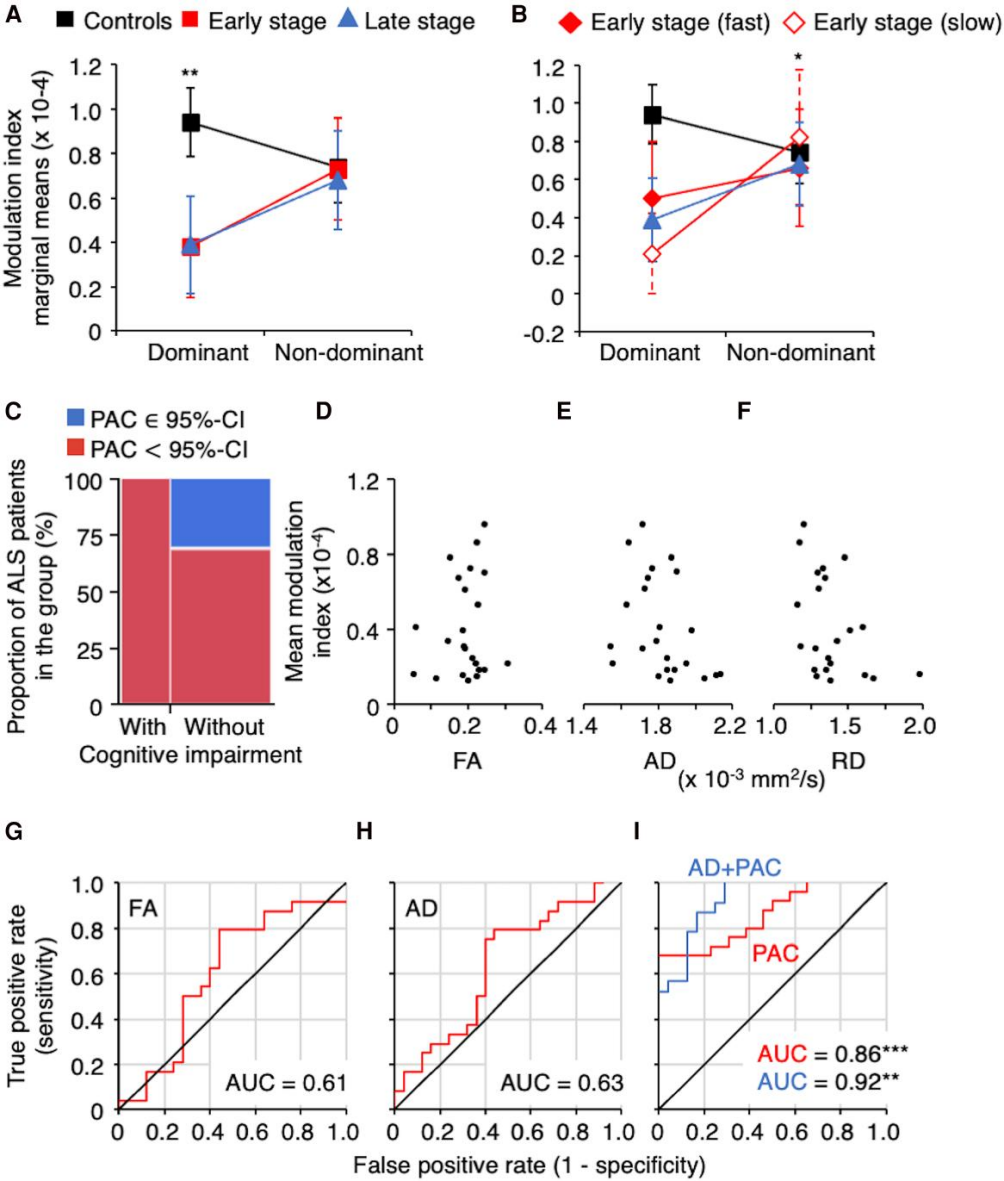
**Links with clinical phenotype**. (**A** and **B**) Marginal means of the theta-gamma PAC modulation index in controls (black) and patients at different disease stages: early stage (≤1 year after disease onset, red) and later stage (>1 year after the first symptom, blue; **A**). Data are presented separately for the dominant hemisphere (left side of the panel) and the non-dominant hemisphere (right side of the panel). Patients in the early stage were further divided into two groups based on their mean progression rate: fast progressors (filled diamonds) versus low progressors (open diamonds; **B**). (**C**) Contingency table illustrating the proportion of patients with amyotrophic lateral sclerosis (ALS), with (left part) or without (right part) cognitive impairment according to their ECAS total score and whether their mean modulation index of theta-gamma PAC on the dominant hemisphere was within the limits of (∈inblue) or strictly inferior to (< in red) to the lower limit of the 95% confidence interval of theta-gamma PAC modulation index in the control group. (**D–F**) Mean modulation index of theta-gamma PAC on the dominant hemisphere in patients with ALS plotted against the fractional anisotropy (FA, **D**), axial diffusivity (AD, **E**) and radial diffusivity (RD, **F**) of the dominant-side corticospinal tract at the brainstem level. (**G–I**) Receiver operating characteristic (ROC) curve illustrating the diagnostic ability of FA (**G**), AD (**H**), and PAC alone (in red) or combined with AD (in blue, **I**). The *y*-axis represents the true positive rate (sensitivity), while the *x*-axis represents the false positive rate (1—specificity). The diagonal black line represents a model with no discriminative power (random guessing). The area under the curve (AUC) quantifies the overall ability of the model to correctly classify positive and negative cases, where an AUC value closer to 1 indicates better performance. *N* = 24 patients with ALS. Linear mixed model: Group (Controls versus ALS), *F*(2,48) = 5.9964, *P* < 0.01. ROC analysis for combined PAC and AD, Chi^2^ = 28.49 for PAC (*P* < 0.0001) and 8.73 for AD (*P* < 0.01). *P < 0.05, ** *P* < 0.001.

Patients were selected based on their motor functions in hand muscles. However, the time since the first symptom was heterogeneous within the group (Table 1). The modulation index of theta–gamma PAC was thus compared between dominant and non-dominant hemispheres (C_d_ and C_n_ electrodes) in controls and patients according to their disease stage (time since onset ≤ 1 year for early stage versus > 1 year for late stage; Table 1). The model (adjusted r^2^ = 0.39) revealed a significant group effect [*F*(2,48) = 5.9964, *P* < 0.01, f^2^ = 0.35] and a significant interaction between group and hemisphere [*F*(2,48) = 6.3523, *P* < 0.01, f^2^ = 0.35], reflecting the difference between controls and patients, further confirming the difference between controls and patients and between brain hemispheres. *Post hoc* comparisons revealed no significant difference between the two subgroups of patients (*P* = 0.88, d = 0.1; Fig. 4A).

Since we previously reported that the mean modulation index of theta-gamma PAC in the present cohort was significantly linked to the disease progression,^21^ the model was recalculated taking the mean progression rate into account (according to the median progression rate). The patient group was divided into three subgroups: five patients at early stage were classified as slow progressors, seven as fast progressors and all patients at the later stage were classified as slow progressors. The model (adjusted r^2^ = 0.46) revealed significant differences between groups [*F*(3,47) = 3.9717, *P* < 0.02, f^2^ = 0.37], hemispheres [*F*(1,48) = 6.0645, *P* < 0.02, d = 0.12] and significant interaction between both factors [*F*(3,47) = 5.0830, *P* < 0.01, f^2^ = 0.37]. *Post hoc* comparisons revealed a significant difference between hemispheres only in patients at the early stage with slow progression (*P* < 0.05, d = 1.16); no statistically significant differences were found in controls (*P* = 0.051, d = 0.41), patients at the late stage with slow progression (*P* = 0.052, d = 0.98) and patients at the early stage with fast progression (*P* = 0.41, d = 0.71; Fig. 4B). Collectively, these results indicate no significant differences between early and late-stage patients with slow progression. However, early-stage patients with slow progression exhibited the most asymmetrical coupling.

Since altered theta–gamma PAC has been particularly observed in patients with cognitive impairment,^24,62^ and that 30–40% of the patients of our cohort had ECAS scores below the cut-off (77/100 and 105/136 for ALS-specific and total ECAS scores, respectively; Table 1),^31^ we investigated the possible links between ECAS scores and altered theta–gamma PAC. First, the correlation between PAC on the dominant hemisphere with ALS-specific and total ECAS scores was not significant [correlation with (i) ALS-specific score = 0.19, 95% confidence interval (CI) from −0.23 to 0.55, *P* = 0.36, r^2^ = −0.006; (ii) total score = 0.19 (−0.23 to 0.55), *P* = 0.38, r^2^ = −0.009]. Second, the proportion of patients with or without cognitive impairment (total ECAS score) and with altered or unchanged PAC (according to CI limits in controls) was statistically significant (Chi^2^ = 4.69, *P* < 0.05, V = 0.31); altered PAC being particularly observed in patients with cognitive dysfunctions (Fig. 4C).

The link between PAC alteration and the clinical phenotype was further explored by considering both behavioural (ALSFRS-R) and cognitive (ECAS) assessments, along with the extent of UMN versus LMN involvement (Table 1). In this cohort of patients, we found only a trend suggesting that the ECAS score decreased with ALSFRS-r [correlation = 0.26 (−0.15 to 0.59), *P* = 0.21, r^2^ = −0.03].^63^ As previously reported,^63^ we observed a greater proportion of patients with predominant UMN involvement exhibiting cognitive impairment (44%) compared to those with predominant LMN involvement (27%) or the classical form (20%). However, the proportion between groups was not significantly different (Chi^2^ = 1.08, *P* = 0.58, V = 0.11).

Lastly, we performed multiple correspondence analysis to evaluate the link between PAC, cognitive and behavioural impairments along with UMN involvement. The Chi^2^ test indicated no statistically significant association (Chi^2^ = 5.46, *P* = 0.36, V = 0.27). This analysis revealed only one significant dimension, with the most influential parameter being altered ECAS score.

### ROC analysis

We tested the possible link between PAC and DTI metrics at the brainstem level on the dominant hemisphere (Fig. 4D–F). Pearson's correlation analyses did not reveal any significant association between PAC and FA [correlation = 0.13 (−0.29 to 0.52), *P* = 0.54, r^2^ = 0.02; Fig. 4B], AD [correlation = −0.39 (−0.69 to 0.03), *P* = 0.07, r^2^ = 0.11; Fig. 4E], or RD [correlation = −0.31 (−0.64 to 0.12), *P* = 0.15, r^2^ = 0.05; Fig. 4F].

ROC analyses were conducted to evaluate the diagnostic performance of DTI metrics in the dominant corticospinal tract at the brainstem level and PAC in the dominant brain cortex. FA did not significantly predict ALS status in our cohort (Chi^2^ = 1.52, *P* = 0.22, V = 0.17), with mean area under the curve (AUC) of 0.609 [CI: (0.607–0.612); Fig. 4G], mean sensitivity of 0.57 (0.48–0.66) and mean specificity of 0.53 (0.45–0.61). ROC analysis for RD yielded similar results [Chi^2^ = 2.75, *P* = 0.09, V = 0.24, AUC = 0.617 (0.613–0.619), mean sensitivity = 0.56 (0.47–0.66), mean specificity = 0.54 (0.46–0.62)]. Among the DTI metrics, AD provided the best stratification [Chi^2^ = 3.71, *P* = 0.054, V = 0.28, AUC = 0.638 (0.635–0.641), mean sensitivity = 0.58 (0.49–0.66), mean specificity = 0.55 (0.47–0.63); Fig. 4H]. In contrast, PAC significantly stratified both groups (Chi^2^ = 25.75, *P* < 0.0001, V = 0.71) and demonstrated substantially higher mean AUC of 0.858 (0.856–0.860; Fig. 4I), better mean sensitivity of 0.69 (0.60–0.77) and mean specificity of 0.67 (0.58–0.76). Combining PAC and AD resulted in even better stratification [Chi^2^ = 28.49 for PAC and 8.73 for AD, with corresponding *P* < 0.0001 and < 0.01, V = 0.80 and 0.42, AUC = 0.926 (0.924–0.928), mean sensitivity = 0.72 (0.63–0.81), mean specificity = 0.69 (0.60–0.78); Fig. 4I]. Adding RD and FA did not alter the results, but the Chi^2^ for AD was no longer significant.

## Discussion

This study demonstrates that the mean modulation index of theta–gamma PAC can be specifically altered at the level of central EEG electrodes, located over the handedness-related dominant primary sensorimotor cortex in patients with ALS, without corresponding changes in power bands or PAC between other frequency pairs. The electrophysiological changes were accompanied by changes in MRI diffusion metrics within the same brain hemisphere. Particularly, FA in the corticospinal tract measured at the brainstem level was greater in the group of patients on the dominant hemisphere, compared to controls. However, no significant association was found between PAC and FA modifications. Lastly, the mean modulation index of theta–gamma PAC was particularly altered in patients with cognitive impairment, and it showed better performance to stratify patients from controls, all the more so when combined with AD.

### Structural alterations in the dominant hemisphere in ALS

Brain changes in ALS encompass both structural and functional modifications, primarily and initially in the motor cortex, where UMN degeneration occurs.^64,65^ Structural alterations involve thinning of the motor cortex, loss of pyramidal neurons and widespread degeneration of white matter tracts, most notably the corticospinal tract.^66,67^ A recent study^68^ using machine learning identified distinct MRI signatures that correspond to four different ALS phenotypes. However, the absence of grey matter features in this classification and the lack of significant differences between controls and patients suggest either a primary axonopathy (rather than neuronopathy) or an insensitivity of grey matter measures in patients with predominant UMN involvement.^68^ DTI has indeed proven more effective in revealing brain structural changes, characterized by reduced FA in the white matter, particularly at the level of the internal capsule, indicating disrupted axonal integrity within the motor pathway.^69^ Most of the DTI metrics selected for corticospinal tract classification came from the right hemisphere, underscoring the asymmetrical hemispheric involvement in ALS.^70-72^ This aligns with another study reporting left-lateralized corticospinal tract impairment in right-handed patients.^73^ However, this lateralization of structural changes in the brain does not appear to necessarily correlate with the side of the body showing the initial motor symptoms. Indeed, a link between handedness and the first affected side has only been observed in patients whose initial symptoms involved the upper limbs.^74^

The present study did not reveal substantial differences in brain structures between patients and controls. However, subtle differences were observed in specific regions. The lack of significant changes in the grey matter of the primary sensorimotor area (cortical thickness) and in the overall microstructure of the corticospinal tract (diffusion MRI) is likely related to the disease stage. The mean ALSFRS-r score of 39.8 ± 3.9 (standard deviation) indicates that the ALS group was, on average, at an earlier stage of the disease compared to groups where significant alterations in diffusion metrics were observed. Specifically, significantly reduced FA and increased RD were reported in patient groups with mean ALSFRS-r score of 32.2 ± 8.6 but not when the mean score was 36.0 ± 7.4.^75^ However, potential heterogeneity among patients across the different cohorts may have compromised the detection of significant differences at the group level. Interestingly, the handedness-related brain asymmetry observed here in controls was less pronounced in patients: FA was particularly more symmetrical between hemispheres in ALS patients compared to controls. Several studies in healthy right- and left-handers have reported similar hemispheric asymmetry at the cortical level.^76-78^ Regarding diffusion metrics, heterogenous results have been reported, showing asymmetries in both directions (left or right) or no significant asymmetry.^79^ DTI results are influenced by multiple factors, including methodological choices (acquisition protocol, analysis pipeline), biological variability (inter-individual differences, functional specialization, neurodevelopment and aging) and the properties of surrounding tissue, which may explain the heterogeneity of findings across studies. The most important aspect of the present study is that when asymmetries were observed, they were in the same direction in both groups and consistent across diffusion metrics (FA, AD and RD). However, more symmetrical DTI results between hemispheres were observed in the ALS group, due to higher FA and consistently lower diffusivity in the dominant hemisphere compared to controls.

To date, increased FA in ALS has only been reported in spinal grey matter at the presymptomatic stage in *Sod1^G93A^* preclinical model.^80^ In human patients, increased FA has been reported in Parkinson's disease^81^ and in semi-acute mild traumatic brain injury.^82^ Such an increase is commonly attributed to higher axonal density, which may contribute to compensatory mechanisms to preserve neural functions.^83,84^ In ALS preclinical model, increased axonal density has been accompanied by elevated levels of aquaporin markers, which regulate membrane permeability.^80^ Interestingly, in traumatic brain injury, the transient increase in FA has been attributed to cytotoxic oedema, involving changes in ionic homeostasis and consecutive alterations in the ratio of intra and extracellular water.^82^ Increased FA is also associated to gliosis in grey matter,^85^ a phenomenon also reported in ALS.^86^ The fact that the present study revealed those changes at the most caudal parts of the corticospinal tract within the brain is likely related to compensatory mechanisms with higher axonal density preceding distal axonopathy in ALS.^80,87^ The greater FA particularly observed at the brainstem level may be also linked to the ALS-related sensitivity of surrounding nuclei and grey matter intermingled with the white matter (bulbar LMNs, breathing centres). Regardless of the origin of the higher FA in the dominant hemisphere, the key finding of the present study is the identification of structural changes in the dominant hemisphere, consistent with altered PAC at the cortical level.

### Theta–gamma phase–amplitude coupling: a tool for assessing cortical dysfunction in ALS

Cortical hyperexcitability is detected at presymptomatic stage and is considered a key factor contributing to UMN loss in ALS.^9^ Thus, it is regarded as a relevant early diagnostic biomarker in ALS, playing a central role in its identification.^88^ Cortical excitability is highly dependent on a variety of mechanisms, among which UMN intrinsic excitability and proper balanced excitatory and inhibitory afferent inputs.^89^ TMS is an effective technique for assessing cortical excitability, which has enabled the identification of two key dysregulated processes in ALS: reduced intracortical inhibition and enhanced intracortical facilitation.^90^ The first process involved GABAergic interneuron dysfunction, and the second is characterized by enhanced UMN outputs (increased excitatory glutamate transmission), which plays a central role in inhibition/excitation imbalance at cortical level.^91^ However, the significant number of patients who do not respond to TMS at early disease stage (∼25%)^92^ and the increase in non-responders as the disease progresses pose major limitations for its use in clinical trials.

There is a growing interest on EEG biomarkers in ALS.^7,93^ Recently, we reported altered PAC between theta and gamma oscillations, both in preclinical models and symptomatic patients (from the same cohort as in the present study).^21^ PAC alteration suggests impaired communication between neural networks, consistently with ALS-related cortical connectivity changes evaluated using EEG and magnetoencephalography.^16,94^ Interestingly, four ALS phenotypes have been identified by studying EEG connectivity,^94^ and the possible link with the four stages established using DTI remains to be determined. Previous studies have also shown a decrease in power across all EEG resting-state frequency bands in ALS patients, who were likely more functionally impaired (ALSFRS-r scores ranging from 10 to 38 across studies) compared to those included in this study.^95-100^ The majority of these alterations have been observed in central channels (C_3_, C_4_, C_z_), with a particular reduction in alpha band power at the C_3_ electrode found to correlate with the degree of bulbar impairment.^100^ Changes in power spectra strongly influence coupling assessment,^60^ but this cannot account for the altered PAC in the present group of patients. Instead, our results indicate that the PAC modulation index is likely a more sensitive marker than spectral power for detecting cortical dysfunction. Moreover, PAC was only altered between theta and gamma oscillations in patients, which might be linked to the role of this coupling in motor skill and its modulation by GABAergic inhibition in the primary motor cortex.^101^ However, it is important to note that the peak activity in the theta band often overlapped with the peak activity in the alpha band. Therefore, future studies should consider evaluating PAC by accounting for the peak activity within the combined theta–alpha band. Additionally, investigating beta-gamma PAC under conditions where the commonly recognized sensorimotor beta rhythm is optimized e.g. movement preparation and execution^102^ or in response to sensory inputs,^103,104^ would be highly valuable.

The brain's capacity to generate cross-frequency coupling depends on synaptic interactions between inhibitory and excitatory neuronal populations. Consequently, any imbalance between excitation and inhibition could disrupt cross-frequency coupling between brain oscillations.^105,106^ This coupling of brain rhythms is recognized as a potential mechanism that allows local and global cortical networks to synchronize and communicate, and it is associated with various cognitive processes, such as memory and sensory processing.^18^ Among the different cross-frequency coupling methods, PAC has recently garnered increasing attention in neurodegenerative disorder research since it was found altered in several conditions, including Alzheimer's disease, frontotemporal dementia, and Parkinson's disease.^23,25,107^ In this study, a specific alteration in theta–gamma PAC was identified in the dominant primary sensorimotor cortex. Previous research has shown that PAC is a valuable tool for lateralizing and localizing epileptic foci.^108^ Furthermore, in early-stage ALS, there is a concordance between the side of symptom onset, the spread of muscle weakness, and handedness in patients with upper-limb onset ALS, which, as reported in the preceding section, is accompanied by lateralized structural defects.^71,73,74^ In addition, our results are also consistent with findings in patients with mild cognitive impairment, exhibiting specific alteration in theta-gamma PAC.^24,62^ Indeed, ALS patients can exhibit mild cognitive impairment, which often precedes motor symptoms,^109^ and we found theta–gamma PAC specifically altered in patients with altered ECAS score. Interestingly, ALS-related cortical hyperexcitability assessed using TMS is particularly associated with cognitive impairment and is predictive of the cognitive phenotype.^110,111^ Consistently, our study revealed a specific link between PAC alteration and cognitive impairment, likely due to the relationship between PAC and cortical excitability.^17,18,21^ However, the connection between PAC, clinical phenotype, and hyperexcitability whether assessed using validated paired-pulse TMS^110,111^ or TMS-EEG protocols^112^ should be further explored in larger cohorts with a balanced representation of the different phenotypes.

The role of PAC in higher-order cognitive functions, such as language, attention, and fluid intelligence, raises the question of whether reduced PAC is a consequence of the disease or a reflection of natural variations in intelligence. Studies have demonstrated that stronger coupling between certain frequency bands, particularly beta–gamma coupling, is associated with superior cognitive abilities.^113^ However, our results did not reveal any differences in this coupling. Furthermore, in studies on Alzheimer's disease and mild cognitive impairment,^24,62^ control groups were thoroughly evaluated for higher-order cognitive functions; yet, a decrease in PAC was still observed in the pathological groups. Similarly, PAC reductions have also been found reduced in ALS preclinical models.^21^ Therefore, the alteration of PAC in ALS is likely linked to the pathology itself. Nonetheless, the sociocultural level of participants should be carefully considered in future studies. Overall, PAC appears to be a fairly reliable marker for detecting brain dysfunctions in neurodegenerative diseases, although it should not be considered a pathology-specific biomarker. It would be valuable to assess PAC in larger cohorts, including patients with various neurodegenerative diseases affecting both cognitive and motor functions, to evaluate its potential in stratifying patient populations.

EEG is known for its remarkable temporal resolution but relatively low spatial resolution. Performing an accurate EEG source localization analysis^114^ would be beneficial to confirm the local dysfunction at the level of the primary sensorimotor cortex. However, we did not observe any changes in the nearest electrodes (C_z_, P_z_, F_z_), and altered PAC limited to the primary sensorimotor cortex at an early disease stage is consistent with ALS pathophysiology.^115^ Moreover, the majority of patients presented with bulbar or lower limb onset (17 out of 26, Table 1), which may explain the lack of a significant association between PAC alterations and the site of onset.^21^ Investigating a larger number of patients with upper limb onset could provide further insight into a potential link between PAC alterations and onset on the dominant side.^74^

Our primary objective was to propose a biomarker that could be easily implemented in clinical practice using a simple EEG setup. Identifying a biomarker at the electrode level provides a straightforward and practical approach for assessing cortical dysfunction in clinical settings. Additionally, previous studies in Alzheimer's disease, dementia and mild cognitive impairment have demonstrated the strong effectiveness of theta-gamma coupling in stratifying patients.^24,62,107^ Accordingly, the ROC analysis demonstrates that the mean modulation index of theta–gamma PAC had excellent diagnostic performance in distinguishing patients from controls and provides valuable additional information to DTI metrics. Our results in patient subgroups suggest that PAC could be useful for evaluating brain dysfunction throughout the disease course. However, these findings need to be confirmed in larger cohorts. The simplicity of the experimental setup for data collection, along with the high sensitivity and specificity of this metric, establishes theta–gamma PAC as a strong candidate to detect cortical dysfunctions in ALS and monitor progression.

### Conclusions and perspectives

We have previously demonstrated that an imbalance between cortical excitatory and inhibitory processes in ALS was associated with neural uncoupling, as assessed through EEG and electrocorticogram recordings in patients and preclinical models, respectively.^21^ In this study, we provide further evidence for this by showing that altered PAC in the dominant hemisphere is accompanied by structural reorganization on the same side, as revealed by validated MRI biomarkers. The absence of correlation between these changes suggests that the mechanisms are likely independent, with one potentially reflecting compensatory adaptations to maintain corticospinal function, while the other indicates cortical dysfunction, both preceding significant neurodegeneration. Moreover, the current findings underscore the high sensitivity and specificity of theta–gamma PAC, reinforcing its potential as a valuable tool for detecting cortical dysfunction in ALS. However, these results require validation through larger-scale studies involving a more extensive cohort of patients at different disease stages to confirm PAC ability to classify patients. Additionally, as indicated by our previous analyses linking PAC to disease progression,^21^ further research is needed to evaluate its predictive value in identifying patient phenotypes. Furthermore, we have shown that PAC is altered at early presymptomatic stages in murine models.^21^ To explore whether similar PAC changes occur at presymptomatic stages in humans, we are currently conducting a new study involving relatives of familial ALS cases linked to the *C9orf72* gene mutation.^116^ In conclusion, the present groundbreaking study establishes resting-state EEG and PAC analysis as a novel quantitative neuromarker for functional degeneration in ALS, presenting a promising avenue for improving diagnostic accuracy, clinical assessment and therapeutic strategies.

## Data Availability

The data are not publicly available due to ethical restrictions. The data may be obtained upon request from the corresponding author, subject to approval from Inserm and authorization from the French national ethics committee.
